# Successful Stent Implantation Into the Patent Ductus Arteriosus in Complex Cyanotic Congenital Heart Disease

**DOI:** 10.7759/cureus.56135

**Published:** 2024-03-14

**Authors:** Kunal Bhugaonkar, Kshitij Balwaik, Neha Masne

**Affiliations:** 1 Department of Cardiology, Jawaharlal Nehru Medical College, Datta Meghe Institute of Higher Education & Research, Wardha, IND; 2 Department of Cardiology, Dr. D. Y. Patil Medical College, Hospital and Research Centre, Navi Mumbai, IND

**Keywords:** duct-dependent heart defects, tetralogy of fallot(t.o.f.), paediatric radiology, paediatric clinical cardiology, cyanotic congenital heart disease, tetralogy of fallot with pulmonary atresia, patent ductus artriosus

## Abstract

Birth-associated structural issues with the heart are known as congenital heart disorders or defects. They might alter the heart’s regular blood flow. A 10-month-old female child presented to a tertiary care hospital with symptoms of recurrent cyanotic spells with episodes of desaturation a few months after birth. ECG findings depicted a normal sinus rhythm with a right axis deviation along the right ventricular forces. Two-dimensional echocardiography showed a tetralogy of Fallot with pulmonary atresia with a patent ductus arteriosus from the undersurface of the arch with confluent small pulmonary arteries. A coronary wire was passed through the left subclavian artery, and a 4 × 16 mm stent was deployed successfully. After the procedure, the patient’s saturation improved, and she was extubated on the table. The patient was on heparin for 24 hours and was started on oral aspirin thereafter. This case was discharged on the third postoperative day and was advised to follow up.

## Introduction

One of the most common birth abnormalities is congenital heart disease (CHD). Research on people and animal models has suggested that CHD has a genetic basis. Approximately 400 genes, including structural proteins and transcription factors crucial for the growth, maturation, and formation of the heart, have been linked to CHD. Recent research has demonstrated the critical roles that genes producing cilia-associated proteins, chromatin modifiers, and cilia-transduced cell signaling mechanisms have in the pathophysiology of CHD [1].

One of the earliest internal body parts to form at the time of embryogenesis is the heart. Mesodermal-derived embryonic precursors give birth to the heart progenitor cells in the cardiac crescent in response to Bmp, Fgf, and Wnt signals in the mouse embryos [2]. A linear cardiac tube is created when these cells align and unite along the central line. The cardiac tube is then looped, with the venous pole becoming the atrial appendages and the outside curve of the looped cardiac tube forming the future ventricles [3]. The conotruncal outflow undergoes septation in parallel, from where the pulmonary and aortic arteries arise. The heart’s migratory neural crest cells are crucial for controlling the outflow septation. Wedging of the outflow of the heart cushions to achieve “mitral to aortic valve continuity” mediates a proper tract of the outflows so that there is a correct orientation of the aorta with the left ventricle and the pulmonary artery with the right ventricle [4]. The epithelial-to-mesenchymal development of endocardial heart cells, resulting in outpouchings known as endocardial cushions, is what mediates the formation of the heart valves. Early in development, these endocardial cushions function as primordial valves, but they subsequently develop to create mature, attenuated valve leaflets [3].

Most of the causes of CHD are unascertained. Merely approximately 15% of CHD cases have an identified etiology. A total of 13 malformation syndromes, which account for 8-10% of CHD, include Turner syndrome, DiGeorge syndrome, Down syndrome, Trisomy 13, and Trisomy 18. Several known chromosomal abnormalities cause these conditions. Single gene defects cause between 3% and 5% of cases of CHD and are frequently linked to noncardiac abnormalities, including Noonan syndrome and Alagille syndrome. It is unclear what causes nonsyndromic CHD. Environmental factors are known to be responsible for about 1.8-2% of total occurrences of CHD. Two of the recognized risk factors are phenylketonuria and pregestational diabetes mellitus. Additional risk factors that have been documented include obesity in mothers, alcohol consumption, infections, and feverish diseases (like rubella or cytomegalovirus infection), usage of specific medications like retinoic acid and thalidomide, and exposure to chemicals during gestation. Men and women present with CHD in different ways as well. In the latter, outflow tract problems such as transposition of the great arteries, aortic valvular stenosis, coarctation of the aorta, and tetralogy of Fallot (TOF) are more prevalent in males. Atrial septal defect type 2 and ventricular septal defect (VSD) are also more common in this population [5].

Timely diagnosis and intervention are essential for a satisfactory denouement. A robust program for the timely diagnosis of CHD and a proper referral system are necessary at the basic community stage. Furthermore, pediatric cardiology and cardiothoracic surgery centers should be set up and supported. An emphasis on treatable CHD and monetary support would assist and improve results [6]. Without proper intervention, only a few survive until one year of age [7].

TOF is one of the most common cyanotic CHDs and is characterized by four cardinal features: VSD; right ventricular outflow tract obstruction (RVOTO), which is often dynamic; overriding of the aorta; and right ventricular hypertrophy. The extent of RVOTO, the relative pressures in the right and left ventricles, and the proportion of the aorta overriding the VSD determine the presentation and severity of this condition [8].

## Case presentation

**Figure 1:**
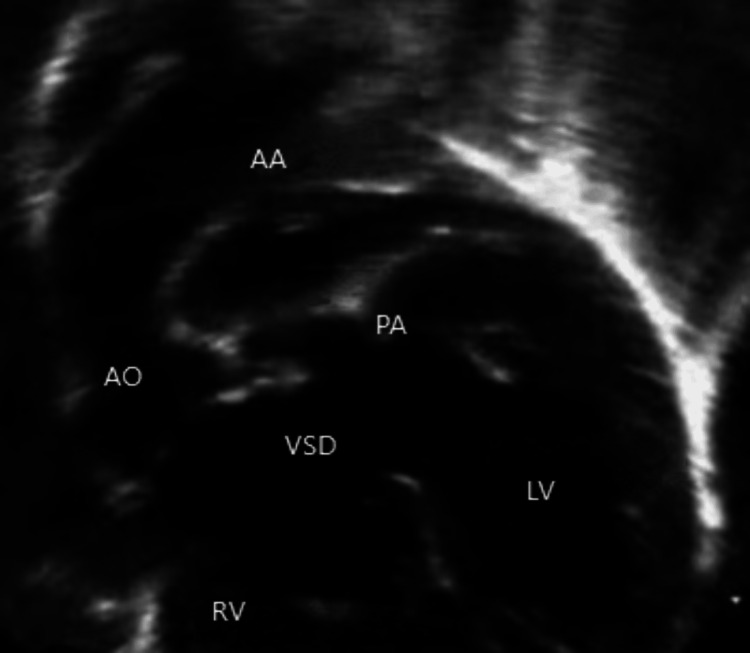
Echocardiogram showing the TOF with PA and PDA from the undersurface of the arch (2D echocardiography view) 2D, two-dimensional; AA, aortic arch; AO, aorta; LV, left ventricle; PA, pulmonary atresia; PDA, patent ductus arteriosus; RV, right ventricle; TOF, tetralogy of Fallot; VSD, ventricular septal defect

A 10-month-old girl child presented with recurring cyanotic spells and desaturation episodes a few months post-birth. She was a full-term normal delivery, asymptomatic in the initial few months. However, at six months, she began experiencing cyanotic spells and had one seizure. On examination, S1 was normal, S2 was a single sound with no murmur, blood pressure was 72/52 mm Hg, and the pulse rate was 124 beats per minute. A two-dimensional (2D) echocardiogram revealed TOF with pulmonary atresia and a patent ductus arteriosus (PDA) originating from the undersurface of the arch with confluent small pulmonary arteries (Figure 1).

Since this was a case of TOF with a right-sided aortic arch, the PDA was necessary for pulmonary circulation. She underwent stenting of the PDA to maintain patency. However, during the aortogram, surprisingly, the PDA, as expected on the right side, could not be visualized (Video 1). Hence, the procedure was abandoned.

**Video 1:** An aortogram is being done to locate the PDA right aortic arch with mirror branching, but the PDA is not visualized PDA, patent ductus arteriosus

**Figure 2:**
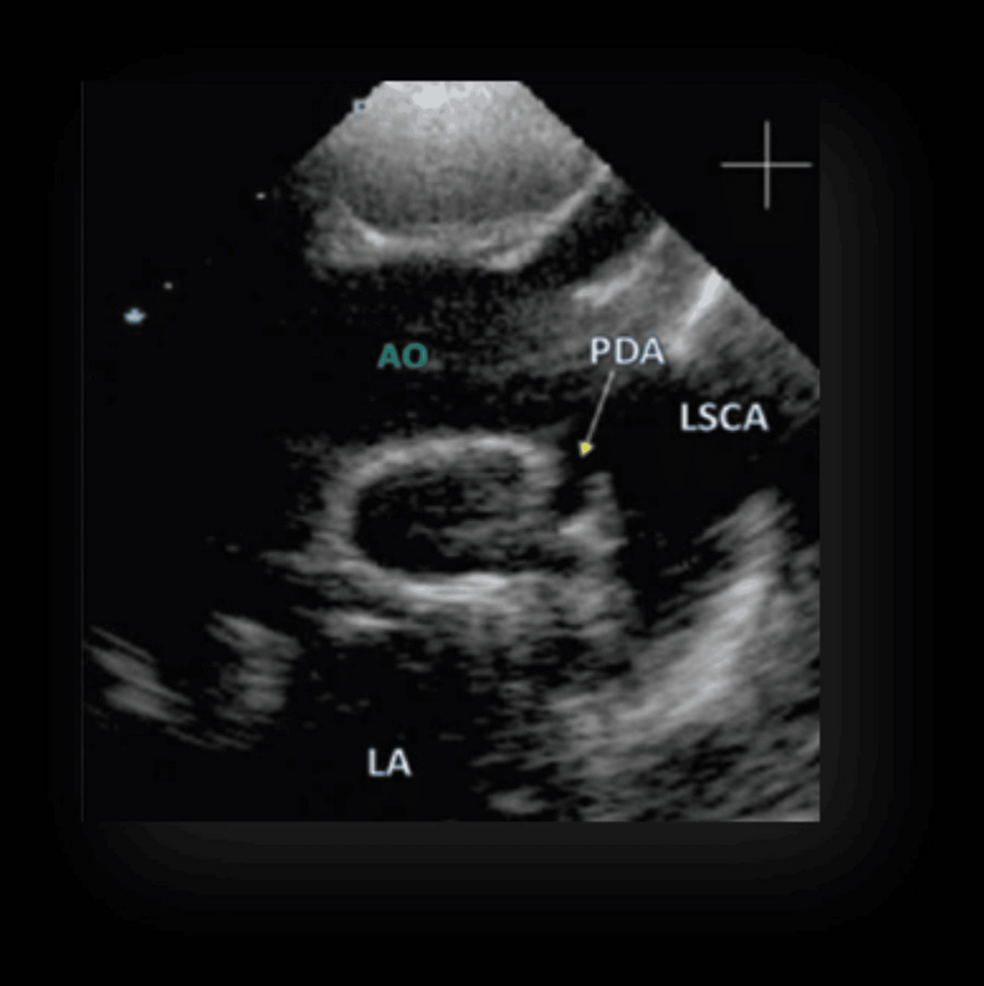
Bedside 2D echo showing persistent PDA 2D, two-dimensional; AO, aorta; LA, left atrium; LSCA, left subclavian artery; PDA, patent ductus arteriosus

However, bedside 2D echo showed persistent PDA (Figure 2).

Next, a selective angiogram of the left subclavian artery was done, which opacified the PDA and showed significant narrowing at the confluence (Video 2).

**Video 2:** Selective angiogram of the LSCA LSCA, left subclavian artery

After this confirmation, a coronary wire was passed through. Subsequently, a 4 × 16 mm stent was deployed successfully (Video 3).

**Video 3:** Stent deployment inside the PDA PDA, patent ductus arteriosus

The patient’s saturation improved post-procedure, and she was extubated on the table. She received subcutaneous heparin (1 mg/kg/dose) for 24 hours, followed by oral aspirin (10 mg/kg/dose). She was discharged after three days of hospital stay and was advised to follow up. PDA stenting is a bridging therapy; a complete repair will be planned once the patient is deemed fit for surgery.

## Discussion

The degree of blood flow restriction to the lungs determines the initial appearance of the TOF. Most newborn patients will exhibit mild-to-moderate cyanosis upon arrival, although they typically do not experience respiratory distress. Rarely, babies with minimal right ventricular outflow tract obstruction at birth may receive a diagnosis only a few months later, when the obstruction strengthens, leading to louder murmurs and newly noticeable cyanosis. Patients with TOF will not show manifestations of cardiac failures, such as failure to thrive, due to severe valvar, sub-valvar, and supra-valvar pulmonary stenosis. Irritability or lethargy is rarely seen in patients with TOF unless they are undergoing a hypercyanotic episode. Individuals newly diagnosed with this condition typically undergo surgery before clubbing can develop, making it highly uncommon in the modern era [9].

Mechanical ventilation, inotropes, and α-agonists such as phenylephrine hydrochloride to enhance pulmonary blood flow are common medical treatments for TOF spells. Prostaglandins can be administered to restore ductal patency in newborns experiencing spells. However, this treatment strategy is not always effective. Patients in need of immediate surgical surgery are those whose conditions are not improving with medical care and stabilization. While these children receive immediate complete repair treatment in some locations, initial surgical intervention with a systemic-to-pulmonary artery shunt and eventual total correction are the treatments provided to these patients in other centers [10].

Stenting the kind of anatomic variation mentioned in the case may present certain difficulties. A single long stent tracking the wire in a long tubular conduit is likely to encounter significant challenges and problems. The ideal procedure is to insert a short stent in the ductus’ distal portion to partially define the ductal constriction, followed by a second stent in the ductus’ proximal section [11].

## Conclusions

TOF has four components, as described above, and management is required for each. In the presence of duct-dependent complex cyanotic CHD, the anatomy of the ductus markedly differs with regard to its origin from the right aortic arch. This current case highlights the importance of being aware of the potential anatomic variants of TOF. So, we should look for an aberrant PDA in the arch of the aorta before contemplating surgery.
